# Psychiatric Diagnoses and Treatment in Nine- to Ten-Year-Old Participants in the ABCD Study

**DOI:** 10.1016/j.jaacop.2023.03.001

**Published:** 2023-03-09

**Authors:** Kelly A. Duffy, Raghu Gandhi, Chloe Falke, Andrea Wiglesworth, Bryon A. Mueller, Mark B. Fiecas, Bonnie Klimes-Dougan, Monica Luciana, Kathryn R. Cullen

**Affiliations:** a University of Minnesota, Minneapolis, Minnesota

**Keywords:** ABCD study, medication, mental health, psychiatric disorders, treatment

## Abstract

**Objective:**

Psychiatric disorders commonly emerge before adulthood. Identification and intervention may vary significantly across populations. A large population-based study was leveraged to estimate the prevalence of psychiatric disorders and treatments and evaluate predictors of treatment in children ages 9 and 10 in the United States.

**Method:**

Cross-sectional data from the Adolescent Brain Cognitive Developmental (ABCD) Study were analyzed. The Schedule for Affective Disorders and Schizophrenia for School-Age Children–Computerized version (K-SADS-COMP) was used to estimate clinical diagnoses, and the Child Behavior Checklist (CBCL) was used to assess internalizing and externalizing psychopathology. Parents reported on prescription medications and other mental health interventions. Prevalence rates of K-SADS diagnoses and treatments were calculated. Logistic regression analyses estimated associations between clinical and sociodemographic predictors (sex at birth, race, ethnicity, income, education, urbanicity) and treatments.

**Results:**

The most common K-SADS diagnoses were anxiety disorders, followed by attention-deficit/hyperactivity disorder, and oppositional defiant disorder. Attention-deficit/hyperactivity disorder and depression diagnoses predicted stimulant and antidepressant medication use, respectively. Bipolar and attention-deficit/hyperactivity disorder diagnoses also predicted antidepressant medications, outpatient treatment, and psychotherapy. The odds of reporting specific treatments varied by sex, ethnic and racial identities, urbanicity, and income.

**Conclusion:**

Expected rates of K-SADS-based psychiatric symptoms are present in the ABCD sample at ages 9 and 10, with treatment patterns broadly mapping onto psychopathology in expected ways. However, important variations in reported treatment utilization across sociodemographic groups were observed, likely reflecting societal and cultural influences. Findings are considered in the context of potential mental health disparities in US children.

Mental health disorders commonly emerge during adolescence, with a median age of onset for any psychiatric illness of 14.^1^ The lifetime prevalence of psychiatric illness during the adolescent period is approximately 50%.^2^ However, compared with adolescence, less is known about the onset and prevalence of psychopathology during preadolescence. Furthermore, while early intervention is important, a minority of youth with mental health disorders receive treatment.^3^ The extent to which preadolescent children with psychiatric symptoms access treatment as well as the nature of those treatments has not previously been characterized on a large scale.

With a sample of 11,878 children recruited at ages 9 and 10 who are being followed prospectively for 10 years, the landmark Adolescent Brain and Cognitive Development (ABCD) Study is poised to advance knowledge about the prevalence of psychiatric diagnoses and treatments during the adolescent transition. Characterizing these patterns in this large, epidemiologically informed sample at baseline is critically important for establishing a foundation of knowledge that will enrich our current understanding of childhood mental health and serve as a reference for clinical trajectories over the course of the study and for adolescence more broadly. Given that an important motivation for the sampling techniques of the ABCD Study was to reflect the sociodemographic variation of the US population,^4^ this project provides an opportunity to examine clinical and sociodemographic factors associated with increased or decreased likelihoods of receiving different treatments for mental health symptoms.

Accordingly, the first aim of the present work was to leverage the ABCD sample to report on the prevalence of symptoms that meet criteria for mental health disorders based on a structured diagnostic interview (collected through both parent and child reports), broadband psychopathology ratings (parent report), and treatments (parent report) in a large, epidemiologically informed sample. The second aim was to examine which clinical and sociodemographic factors were related to the likelihood of children receiving various treatments. To our knowledge, this is the first large-scale analysis to evaluate the prevalence and demographic and clinical correlates of mental health disorders and psychiatric treatments in preadolescent children in the United States using the ABCD sample.

## Method

### Sample

The ABCD Study enrolled 11,878 children living in the United States when they were 9 or 10 years old. Data from the baseline assessment were used in the present analysis. The ABCD sample was recruited primarily via local schools using a probability sampling method in catchment areas determined based on the included study sites. Some participants were recruited via community outreach and referrals. Twins were recruited via birth registries. The recruitment aim was to match national proportions of age, gender, race and ethnicity, socioeconomic status, and urbanicity. Some targets (eg, participants from rural/nonurban areas) were oversampled due to underrepresentation in the catchment population. Additionally, propensity weights were estimated for each participant for inclusion in analyses to better calibrate the ABCD sample distributions to population-level expectations and to minimize bias. Some sources of sampling biases, such as self-selection into the study population by families, are never entirely avoidable, but best attempts were made by the study organizers to minimize these concerns. Detailed information on the recruitment strategy of the ABCD Study has been reported elsewhere^4^ and in Supplement 1, available online.

### Measures

Parents reported their child’s identified age, sex assigned at birth, race, ethnicity, household income, and other demographic characteristics. Descriptive statistics are presented in Table 1. For this analysis, race and ethnicity were categorized as American Indian/Alaska Native, Asian, Black/African-American, Hispanic/Latino/Latina, Native Hawaiian/Pacific Islander, other (a specific category selected by the parent), and White. In line with the ABCD Study’s approach, groups were not mutually exclusive: participants could endorse multiple categories. For subsequent analyses, the American Indian/Alaska Native and Native Hawaiian/Pacific Islander groups were combined to avoid unreliable estimates due to small numbers in the Native Hawaiian/Pacific Islander group. Household income was classified as less than $50,000, between $50,000 and $100,000, and greater than $100,000. Parental education was classified based on whether either parent had received some amount of college education, regardless of whether a degree was obtained. The urbanicity of participants’ primary addresses was classified according to the Census Bureau as either urbanized areas (containing 50,000 or more people in a census tract), urban clusters (between 2,500 and 50,000 people), or rural areas (less than 2,500 people).^5^ Sex assigned at birth was categorized as male or female. Children identified as intersex males (*n* = 4) were coded as male.

**Table 1:** Descriptive Statistics of Sample

Sociodemographic variables	No.	%
Sex at birth		
Female	5,681	47.8
Male	6,196	52.2
Unknown	1	0
Household income		
<50,000	3,223	27.1
≥50,000 and <100,000	3,071	25.9
≥100,000	4,565	38.4
Unknown	1,019	8.6
Parental education		
Any college	10,138	85.4
Unknown	15	0.1
Race		
American Indian/Alaska Native	410	3.5
Asian	751	6.3
Black	2,518	21.2
Native Hawaiian/Pacific Islander	74	0.6
Other	800	6.7
White	8,806	74.1
Unknown	171	1.4
Ethnicity		
Hispanic/Latino/Latina	2,411	20.3
Unknown	10	0.1
Urbanicity		
Urbanized area	9,856	83
Urban cluster	372	3.1
Rural	966	8.1
Unknown	684	5.8

Note: Total sample is 11,878 participants.

Information on the child’s psychiatric symptoms was gathered from children and their parent/guardian using the computerized version of the Schedule for Affective Disorders and Schizophrenia–Present and Lifetime Version (K-SADS-COMP).^6^^,^^7^ While parents/guardians completed all modules, children completed only the mood, social anxiety, generalized anxiety disorder, suicide, and sleep modules. Symptom reports within these modules were then categorized as either meeting or not meeting *DSM-5* thresholds for each diagnosis. Parents/guardians also reported the child’s behavior over the past 6 months on the Child Behavior Checklist (CBCL).^8^ Sum scores from the broadband scales of internalizing and externalizing problems were analyzed. The *t* score was used to estimate the prevalence of clinically significant internalizing and externalizing problems. Raw scores were used to evaluate associations between CBCL scores and treatments.^8^

Information on prescription medications use of participants was gathered from the Parent Medications Survey Inventory, modified from PhenX.^9^ The parent/guardian identified the names of all prescription medications taken by the child in the last 2 weeks as well as the dosage, frequency, and time when last taken. Reported medications were coded by experts with the matching RxCUI (machine-readable code or unique identifier for the RxNorm system^10^) number from the US Food and Drug Administration. We matched the RxNorm identifiers of the medications to classes within the Anatomical Therapeutic Chemical classification using the National Library of Medicine RxClass Application Program Interface.^11^ The medications reported, their categories, and associated Anatomical Therapeutic Chemical codes are shown in Table S1, available online. As part of the K-SADS-COMP background questions, parents reported on levels of care their child had received (eg, outpatient treatment, partial hospitalization) and specific types of treatment (eg, psychotherapy). Options describing treatment levels were not mutually exclusive, eg, outpatient treatment could encompass medication visits and psychotherapy.

### Statistical Methods

To better represent US population demographics of 9- and 10-year-olds, all analyses incorporated poststratification weights to match the distribution of sociodemographic factors (age, sex, and race/ethnicity) in the US Census Bureau’s American Community Survey as described by Heeringa and Berglund.^12^ Unweighted results for all analyses were similar and are provided in Tables S2 through S5, available online. The prevalence of psychiatric diagnoses (estimated by the K-SADS-COMP), as well as clinically-significant internalizing and externalizing problems according to CBCL *t* scores (determined as *t* scores > 65), was calculated using weighted cross-tabulation. Prevalence was also calculated for all treatment categories.

To better understand associations between psychiatric interventions and clinical characteristics, adjusted odds ratios were calculated using weighted logistic regression for the association between clusters of diagnoses (eg, depressive disorders, anxiety disorders) and raw CBCL internalizing and externalizing scores with the usage of each medication class and each psychiatric intervention as well as any medication use or other mental health intervention overall. Note that adjusted odds ratios give the unique effect of each variable, holding all other modeled variables constant. Diagnosis clusters were defined by the presence of diagnosis per parent-report K-SADS-COMP or (if available for a given diagnosis) child-report K-SADS, combining current, partial remission and/or past diagnoses. Prevalence rates for individual disorders included in each cluster are in Table S6, available online, and prevalence rates for all interventions within the subset of the sample diagnosed with each cluster are in Tables S7 and S8, available online. Substance use disorder was not included as a predictor, given its low prevalence (0.1%). To better understand how psychiatric interventions varied by sociodemographic characteristics, we conducted weighted logistic regressions to estimate adjusted odds ratios for several sociodemographic variables (sex assigned at birth, household income, parental education, race/ethnicity, and urbanicity) predicting each medication class usage and psychiatric intervention as well as any medication or other mental health intervention generally. Within each logistic regression, significance of the predictors was quantified using the Wald χ^2^ test statistic. Corresponding *p* values were corrected using the Benjamini-Hochberg method to control the false discovery rate.^13^

Although not a central goal of the study, to better contextualize results, we also examined the prevalence of psychiatric diagnoses across the sociodemographic categories and again used adjusted odds ratios to evaluate the extent to which each sociodemographic predictor increased the likelihood of diagnosis for each disorder cluster; see Tables S9 through S12, available online. All analyses were conducted using R^14^ and the “survey” package.^15^

## Results

### Prevalence of Psychiatric Diagnoses and Interventions

The prevalence of psychiatric diagnoses as measured by the parent- and child-report K-SADS-COMP is reported in Table 2. For diagnosis clusters rated by both child and parent, the rate of depressive disorders was slightly higher in parent reports, while the rate of bipolar disorders was lower in parent reports. For the broad category of anxiety disorders, the rate was much higher for parent reports, but notably the child-report K-SADS included only 2 of 7 measured anxiety disorders (see Table S6, available online). The remaining diagnoses were assessed only via parent report, with the most common diagnoses being attention-deficit/hyperactivity disorder (ADHD) (18.8%) oppositional defiant disorder (ODD) (14%) and obsessive-compulsive disorder (9.6%). Table 2 also reports the prevalence of clinically significant internalizing and externalizing symptoms.

**Table 2:** Prevalence of Clinically Significant Symptom Reports According to Schedule for Affective Disorders and Schizophrenia for School-Age Children–Computerized version (K-SADS-COMP) and Child Behavior Checklist (CBCL) Reports in the ABCD Sample

	Parent report, weighted %	Child report, weighted %
**Prevalence of psychiatric disorders according to symptom reports on K-SADS-COMP**		
Depressive disorders	6.5	5.4
Bipolar disorder	7.4	9.6
Anxiety disorders	33.7	1.3
Psychotic disorders	0.6	—
OCD	9.6	—
Eating disorders	0.9	—
ODD	14	—
Conduct disorder	3.3	—
PTSD	2.1	—
Substance use disorder	0.1	—
ADHD	18.8	—
**Prevalence of clinically significant CBCL internalizing and externalizing total scores**		
CBCL internalizing *t* score >65	7.7	—
CBCL externalizing *t* score >65	5.0	—

Note: Total sample is 11,878 participants. Percentages are weighted to match American Community Survey demographic proportions. Unweighted values are in Table S2, available online. For the diagnostic clusters reported in subsequent tables, the depression, anxiety, and bipolar clusters combine parent and child reports of depressive disorders, anxiety disorders, and bipolar disorders, respectively, and the disruptive behavior disorders cluster combines the ODD and CD diagnoses. Note that for the anxiety disorders cluster only, the specific disorders included in parent and child reports differ: parent report evaluates symptoms of social anxiety disorder, selective mutism, panic disorder, agoraphobia, separation anxiety disorder, specific phobia, and generalized anxiety disorder, while child report evaluates only symptoms of social anxiety disorder and generalized anxiety disorder. See Table S6, available online, for more information. ADHD = attention-deficit/hyperactivity disorder; OCD = obsessive-compulsive disorder; ODD = oppositional defiant disorder; PTSD = posttraumatic stress disorder.

Table 3 reports parent-reported treatment prevalence. Overall, 9.7% of children received some type of psychiatric medication. Stimulants were the most commonly reported medication (8.4%), followed by antidepressant (1.9%) and antipsychotic (0.6%) medications. Overall, 15.4% received at least one mental health intervention.

**Table 3:** Prevalence of Interventions

Intervention	Weighted sample, %
Medications^a^	
At least one class of medications	9.7
Stimulants	8.4
Antidepressants	1.9
Antipsychotics	0.6
Sedatives	0.2
Anxiolytics	0.2
Other interventions for mental health	
At least one intervention	15.4
Outpatient^b^	9.1
Partial hospitalization	0.2
Inpatient	0.6
Psychotherapy	4.7
Other	2.9

Note: Total sample is 11,878 participants. Percentages are weighted to match American Community Survey demographic proportions. Unweighted values are in Table S3, available online.

a Medication classes defined using the Anatomical Therapeutic Chemical Classification system.

b Note that categorizations of treatment levels were based on parental discretion and not mutually exclusive; outpatient treatment in particular is a level of care that may potentially encompass several types of treatment, including medication visits and psychotherapy.

### Clinical Correlates of Psychiatric Interventions

Table 4 reports the adjusted odds ratios representing the extent to which clinical indexes increase the likelihood of receiving different medications and other mental health interventions. Higher CBCL externalizing symptoms and K-SADS-threshold ADHD diagnosis were associated with increased odds of using at least one type of medication, while both internalizing and externalizing symptoms, as well as a number of diagnoses, increased the odds of at least one other mental health intervention. Higher externalizing symptoms were associated specifically with increased odds of stimulant, antipsychotic, and anxiolytic usage as well as outpatient, inpatient, and “other” treatments. Higher CBCL internalizing symptoms were associated with higher odds of antidepressant usage, outpatient treatment, and psychotherapy. Reaching the K-SADS diagnostic threshold for ADHD increased the odds of antidepressant usage, outpatient treatment, and psychotherapy as well as stimulant usage. Children reaching the K-SADS diagnostic threshold for disruptive behavior disorders (ODD and conduct disorder) were more likely to receive outpatient treatment and psychotherapy. The presence of depression or bipolar K-SADS-threshold diagnoses were each associated with greater odds of antidepressant usage, outpatient treatment, and psychotherapy. Children reaching the diagnostic threshold for eating disorders and psychotic disorders were less likely to use anxiolytic medications. Children reaching the diagnostic threshold for posttraumatic stress disorder were more likely to receive outpatient treatment. In general, these patterns conform to expectations regarding alignments between treatments and reported symptoms.

**Table 4:** Clinical Predictors of Interventions: Adjusted Odds Ratios for Medication and Treatment by Schedule for Affective Disorders and Schizophrenia for School-Age Children–Computerized version (K-SADS-COMP) Diagnosis and Child Behavior Checklist (CBCL) Internalizing and Externalizing Scores

	At least one medication	Stimulants	Antidepressants	Antipsychotics	Sedatives	Anxiolytics	At least one other intervention	Outpatient^a^	Partial hospitalization	Inpatient	Psychotherapy	Other
Intercept	0.03∗∗∗	0.03∗∗∗	0∗∗∗	0∗∗∗	0∗∗∗	0∗∗∗	0.05∗∗∗	0.03∗∗∗	0∗∗∗	0∗∗∗	0.02∗∗∗	0.01∗∗∗
CBCL internalizing (raw scores)	1	0.99	1.06∗	0.97	1.09	1.06	1.05∗∗∗	1.03∗∗	1.03	0.99	1.06∗∗∗	1.03
CBCL externalizing (raw scores)	1.06∗∗∗	1.05∗∗∗	1.03	1.13∗∗∗	1.01	1.1∗∗	1.05∗∗∗	1.06∗∗∗	1.08	1.11∗∗	1	1.04∗
Depression	1.17	0.95	1.85∗	2.17	1.48	2.22	1.48∗∗∗	1.42∗	1.17	1.88	1.48∗∗	0.95
Bipolar	1.3	1.2	1.7∗	1.34	2.47	0.72	1.54∗∗∗	1.57∗∗	0.82	0.72	1.58∗∗	1.29
Anxiety	1.17	1.22	1.01	1.94	0.94	3.48	1.11	0.98	1.42	1.83	1.19	1.14
Psychosis (parent report)	1.33	1.17	0.77	2.3	3.33	0∗∗∗	1.04	1.24	1.77	2.1	0.9	0.58
OCD (parent report)	1.15	1.18	1.3	1.35	0.71	0.71	1.15	1.01	1.43	0.61	1.15	1.42
Eating disorder (parent report)	0.7	0.64	1.16	1.11	3.58	0∗∗∗	1.33	1.12	2.38	3.15	1.31	1.26
ODD/CD (parent report)	1.17	1.18	1.59	1.55	1.42	0.33	1.96∗∗∗	1.93∗∗∗	1.56	1.97	1.78∗	1.29
PTSD (parent report)	0.99	0.95	0.98	0.6	0.16	0.45	2.57∗∗	2.77∗∗	0.69	2.97	1.28	0.92
ADHD (parent report)	5.08∗∗∗	6.23∗∗∗	2.09∗	1.48	2.3	1.56	1.74∗∗∗	1.59∗∗	2.23	1.65	1.8∗∗	1.32

Note: Results are weighted to match American Community Survey demographic proportions. Unweighted values are in Table S4, available online. Note that adjusted odds ratios of 0 in the table are rounded to zero and do not represent true zero values, but rather reflect that there were no observed instances of the combination of treatment type and clinical diagnosis in the sample. ADHD = attention-deficit/hyperactivity disorder; OCD = obsessive-compulsive disorder; ODD/CD = oppositional defiant disorder/conduct disorder; PTSD = posttraumatic stress disorder.

a Note that categorizations of treatment levels were based on parental discretion and not mutually exclusive, and outpatient treatment in particular is a level of care that may potentially encompass several types of treatment, including medication visits and psychotherapy.

∗ *p* < .05; ∗∗*p* < .01; ∗∗∗*p* < .001; *p* values adjusted for multiple comparisons using the Benjamini-Hochberg method.

### Sociodemographic Correlates of Medication Treatment

Table 5 reports the adjusted odds ratios representing the extent to which categorical sociodemographic predictors increase the likelihood of children receiving specific medications. Holding all other sociodemographic variables constant, relative to participants assigned female at birth, participants assigned male at birth were more likely to use at least one type of medication and to report a mental health intervention generally and specifically more likely to report stimulant, antidepressant, and antipsychotic use as well as outpatient treatment. Compared with children whose families reported annual incomes of less than $50,000, children from families with annual incomes between $50,000 and $100,000 and above $100,000 were less likely to use at least one type of medication or to report another mental health intervention generally and had lower odds of reporting outpatient treatment and stimulant, antipsychotic, and anxiolytic medications in particular. Children from families with incomes above $100,000 were also less likely to report inpatient and partial hospitalization treatments. Children identified by parents as Black had greater odds of receiving stimulant medications than those not identified as Black (note that as race and ethnicity categories were not mutually exclusive, results compare those who were identified as a particular race or ethnicity relative to all those who did not endorse that identity, adjusted for all other variables in the model). Children identified as Asian were less likely to receive inpatient or partial hospitalization treatment. Children identified as “other” race were less likely to use anxiolytic and sedative medications and to receive inpatient treatment. Children identified as Indigenous (ie, Alaska Native, American Indian, Native Hawaiian, and Pacific Islander) had increased odds of using anxiolytic medications. Children identified as White were more likely to use at least one type of medication and to report a mental health intervention generally and specifically more likely to report using antidepressant, stimulant, and antipsychotic medications, as well as outpatient treatment and psychotherapy, than children not identified as White. Children identified as Hispanic/Latino/Latina had lower odds of receiving a mental health intervention generally and specifically of receiving any outpatient treatment. Compared with participants in urban areas, rural residents were less likely to report anxiolytic usage and partial hospitalization treatments, while participants in urban clusters (ie, small towns) were more likely to use antipsychotics. Participants with a parent who reported at least some college education were more likely to report a mental health intervention generally than participants whose parents did not report any college education.

**Table 5:** Sociodemographic Predictors of Interventions: Adjusted Odds Ratios

	At least one medication	Stimulants	Antidepressants	Antipsychotics	Sedatives	Anxiolytics	At least one other intervention	Outpatient^a^	Partial hospitalization	Inpatient	Psychotherapy	Other
Intercept	0.04∗∗∗	0.03∗∗∗	0∗∗∗	0∗∗∗	0.01	0∗∗	0.1∗∗∗	0.07∗∗∗	0.01∗∗	0∗∗	0.02∗∗∗	0.01∗∗∗
Sex at birth: male	2.63∗∗∗	3.06∗∗∗	1.71∗	2.71∗	1.34	1.88	1.41∗∗	1.44∗∗	1.37	1.89	1.23	1.48
Household income: ≥50,000 and <100,000	0.6∗∗	0.62∗∗	0.78	0.5∗	1	0.11∗	0.62∗∗	0.56∗∗	0.34	0.4	0.83	0.77
Household income: ≥100,000	0.53∗∗∗	0.53∗∗∗	0.68	0.24∗	0.58	0.02∗	0.51∗∗∗	0.39∗∗∗	0∗∗∗	0.13∗	0.94	0.69
Parental education: any college	1.29	1.19	4.04	1.21	1.07	1.91	1.6∗	1.32	0.36	1.44	2.26	1.58
Asian	0.54	0.62	0.32	0.2	0.13	0.68	0.72	0.75	0∗∗∗	0∗∗∗	0.69	1.12
Black	1.36	1.5∗	1.23	1.48	0.17	1.02	1.05	1.16	1.1	2.45	0.79	1.23
Indigenous^b^	1.03	1.01	0.88	1.14	0.85	2.98∗	0.92	0.99	0.5	0.94	0.67	0.76
Other race	1.19	1.11	3.16	2.98	0∗∗∗	0∗∗∗	1.12	0.99	2.58	0∗∗∗	0.85	1.83
White	1.73∗	1.84∗	2.36∗	2.12∗	0.32	2.81	1.83∗∗∗	1.85∗∗	1.09	2.04	1.85∗	1.63
Hispanic/Latino/Latina	0.7	0.81	0.33	0.34	0.26	0.2	0.56∗∗	0.43∗	0.26	0.24	0.98	0.77
Urbanicity: urban cluster	1.62	1.51	1.76	3.72∗	1.35	1.46	1.11	1.19	2.09	1.97	0.6	1.76
Urbanicity: rural	1.24	1.28	0.96	1.86	2.84	0∗∗∗	0.81	0.97	0∗∗∗	0.61	0.66	0.57

Note: Results are weighted to match American Community Survey demographic proportions. Unweighted values are in Table S5, available online. Note that adjusted odds ratios of 0 in the table are rounded to zero and do not represent true zero values, but rather reflect that there were no observed instances of the combination of treatment type and demographic characteristic in the sample.

a Note that categorizations of treatment levels were based on parental discretion and not mutually exclusive, and outpatient treatment in particular is a level of care that may potentially encompass several types of treatment, including medication visits and psychotherapy.

b American Indian, Alaska Native, Native Hawaiian, or Pacific Islander.

∗ *p<* .05; *∗∗p<* .01; *∗∗∗ p<*.001; *p* values adjusted for multiple comparisons using the Benjamini-Hochberg method.

## Discussion

This study leveraged the ABCD Study’s large baseline sample of children ages 9 and 10 to estimate the prevalence of mental health disorders and their treatments during this critically important developmental period and evaluated the clinical and sociodemographic correlates of treatment types. Generally, prevalence rates of psychiatric disorders at the ABCD baseline align with expectations based on prior youth studies. Anxiety disorders, ADHD, and ODD were the 3 most prevalent disorders, which coheres with a recent meta-analysis of prevalence studies in youth aged 4 to 18 years.^3^ However, higher rates for all 3 disorders were observed in the ABCD sample. Nearly one-third of ABCD parents reported a past or present anxiety disorder in their child; this is consistent with the National Comorbidity Survey–Adolescent Supplement (NCS-A), which reported a prevalence of 31.9% for anxiety disorders in adolescents aged 13 to 18 years with a median onset age of 6 years,^2^ and with the general National Comorbidity Survey, which suggested that many anxiety disorders have already emerged by preadolescence.^1^ The observed ADHD prevalence rate was higher than some previous estimates, such as a recent Centers for Disease Control and Prevention prevalence estimate of 9.2% for children aged 6 to 11,^16^ but there is some evidence that the prevalence of ADHD is increasing.^17^ Additionally, the reliance on parent report only, without incorporating reports from other informants (eg, teachers), may be inflating the prevalence rates. The K-SADS-based prevalence for ODD was broadly consistent with previous literature indicating a prevalence range from 1% to 16% in children and adolescents^18^ and similar to the NCS-A estimate of 12.6%.^2^ The relatively lower prevalence of depressive disorders, psychotic disorders, substance use disorders, and eating disorders in the baseline ABCD sample compared with adolescent samples such as the NCS-A^2^ is consistent with a later onset of these conditions.^2^^,^^19^ Prevalence rates for obsessive-compulsive disorder and bipolar disorders were higher than reported in prior pediatric studies,^2^^,^^20^ possibly reflecting a limitation of symptom reports from children and parents, which may not agree with clinician evaluation, for these diagnoses.

The ABCD Study provides a unique opportunity to advance understanding of the utilization of mental health services during an age range that may be vulnerable to emergent psychopathology. The 15.4% of ABCD parents reporting their child had received some sort of mental health treatment falls within estimates from prior work: the National Survey of Children’s Health (NSCH) study reported that 10.1% of children and adolescents 3 to 17 years old received treatment from a mental health professional in the last year; the National Survey on Drug Use and Health (NSDUH) study reported 25.9% of adolescents 12 to 17 years old received mental health services in the last year.^21^ Outpatient level of care was 15 times more common than inpatient and 45 times more common than partial hospitalization treatment. The particularly low rates of partial hospitalization could be due to a relative scarcity in the United States of this level of treatment for preteen children.^22^ About 10% of participants were taking at least one psychiatric medication, slightly higher than what has been reported in previous studies spanning a wider age range: the National Health and Nutrition Examination Survey found 6.6% of 3- to 17-year-olds had taken medication for mental health problems in the last 30 days; the NSCH reported 7.8% of similarly aged youth had taken medication in the past year.^21^

We found that a diagnosis of depression increased the odds of antidepressant usage, and a diagnosis of ADHD increased the odds of stimulant medication usage, suggesting an expected pattern for the mapping of psychopharmacological treatment to these symptom domains. Unexpectedly, bipolar disorder diagnosis also predicted antidepressant usage; as antidepressants are relatively contraindicated in bipolar disorder,^23^ this finding raises a question about the validity of the K-SADS-COMP bipolar diagnosis. Our finding that an ADHD diagnosis increased the odds of antidepressant use is consistent with previous findings that nearly 25% of children with ADHD aged 6 to 12 who are prescribed stimulants are concomitantly prescribed other psychotropic medications, including 6.8% to 7.9% who are prescribed selective serotonin reuptake inhibitors.^24^ This may be due to comorbidity with other psychiatric disorders^25^; however, there is also previous work showing that many youth with ADHD, including 26% of those without comorbid diagnoses, take non-ADHD medications both with and without simultaneous stimulant use.^26^

Children assigned male sex at birth were more likely than children assigned female sex to report use of any medication and any other mental health intervention as well as several specific treatments. This supports previous findings that children and adolescents identified as males ages 3 to 17 are more likely to receive numerous types of mental health services, including medication.^21^ In particular, the increased likelihood of stimulant treatment among male children and adolescents has been well documented,^27^^,^^28^ which may be attributed to the higher rate of ADHD diagnosis in those identified as male, documented in the present study (Tables S9-S12, available online) and consistently in the literature.^2^^,^^21^ Male participants were also more likely than participants identified as female to be diagnosed with disruptive behavior disorders, obsessive-compulsive disorder, and bipolar disorders (Table S11, available online), which may explain the higher likelihood of intervention.

Our findings suggest an impact of income on rates of receiving psychiatric treatments. Children from higher-income families were less likely to receive a number of medications, including stimulants and antipsychotics, and less likely to receive various types of other mental health interventions. This pattern is consistent with previous studies, including a recent surveillance report evaluating multiple nationwide US studies, which suggested that as household income increased, children and adolescents aged 3 to 17 were somewhat less likely to have recently taken medication for mental health problems and generally less likely to receive many other types of mental health services.^21^ This pattern is also seen for specific medications and interventions. For example, the increased likelihood of stimulant usage in children and adolescents with lower socioeconomic status has been documented in several (wealthy, Western) countries, including the United Kingdom^29^ and Sweden.^30^ This pattern may stem from several factors. Children from lower income brackets appear more likely to be diagnosed with a number of mental health disorders, as shown in the present study (Tables S9-S12, available online) and prior literature^21^; also, diagnostic status was not controlled for in the current analyses. Additionally, as proposed by previous work,^29^ stimulant use may be higher in children with lower socioeconomic status due to inequitable access to resources needed to offer children additional support that can reduce impairment from ADHD, such as increased educational support.

Urbanicity also impacts rates of psychiatric treatments. Children from the ABCD Study living in rural areas were less likely to use anxiolytics and receive partial hospitalization treatment than children in urban areas, while those in urban clusters were more likely to take antipsychotic medications. While there is little evidence of how urbanicity impacts treatment usage in the literature, the NSCH study showed that children in rural versus urban areas are more likely to use medications for mental health problems.^21^ These findings likely reflect health disparities in access to mental health resources, such as psychotherapy, structured outpatient treatment, and partial hospitalization, in more rural settings.^31^, ^32^, ^33^

Finally, we found that rates of reported treatment utilization varied across racial and ethnic identities, suggesting potential health disparities. Children whose identities included White race were more likely to report any medication use and any other mental health intervention and more likely to receive a number of specific treatments, including stimulants. This is consistent with a recent surveillance report evaluating multiple US studies, which found that youth who identified as White had the highest estimated prevalence of all types of mental health service engagement, including medications.^21^ Children in this sample who identified as Black were also more likely to receive stimulants, holding all other sociodemographic variables constant. This potentially represents a change from prior work showing that Black children were about half as likely to use stimulants for ADHD compared with White children.^34^ Interestingly, while both White and Black racial identities were associated with a greater odds of meeting criteria from the parent-report K-SADS-COMP for ADHD in this sample (Table S11, available online), prior work has suggested that at kindergarten entry, Black children were 70% less likely to have received an ADHD diagnosis than otherwise similar White children.^35^ It is possible that the ABCD Study may be capturing clinically significant ADHD symptoms in children who have not been evaluated and whose diagnoses would be missed by relying on a prior history of a medical diagnosis. While greater access to care may be driving increased likelihood of stimulant use in White children, it is also possible that implicit biases may be contributing to the increased rates of stimulant use in Black children. Recent data suggest that children and adolescents who identify as Asian had the lowest prevalence of engagement in all types of mental health services^21^; similarly, ABCD Study children who identified as Asian were significantly less likely to receive inpatient and partial hospitalization treatment. Our finding that children who identified as Hispanic/Latino/Latina were less likely to receive any nonmedication mental health intervention supports results from the NSCH study that children who identified as Hispanic were less likely to receive professional mental health services than children with some other identities.^21^ Finally, children with Indigenous identities were more likely to be prescribed anxiolytics, although they were not more likely to be diagnosed with anxiety or any other disorder when weighting the sample to American Community Survey proportions (Table S11, available online). While these findings are preliminary due to the small number of children in this category and even smaller numbers of those taking anxiolytics, given the potential risks of this class of medications (eg, potential for long-term dependence) in a historically marginalized population, additional research is warranted.

The findings reported here add to a large body of literature that has recognized the deficits in the US mental health care system that negatively and disproportionately affect children from minoritized communities.^36^, ^37^, ^38^ Several sociocultural issues play a role in the initiation and continuation of mental health treatment.^39^ Minoritized youth face several barriers to effective mental health care, including population barriers (eg, socioeconomic disparities, stigma), provider factors (eg, deficits in cross-cultural knowledge), and systemic factors (eg, services location and organization).^40^ Given this context, more work is needed to implement consensus recommendations for optimal delivery of mental health services for ethnic and racially minoritized populations within the cultural awareness framework.^41^^,^^42^ This will require clinician-related factors (acquiring skills that enable serving populations different from their own) and system factors (eg, changing policies and practices that present barriers to diverse populations). Best practice that fully incorporates cultural awareness, competence, and humility^43^ includes the adoption of culturally specific therapeutic modalities and adaptation of mainstream modalities following assessment of their efficacy with diverse populations.^40^

Several limitations must be noted, regarding both the ABCD protocol and the methodology of this analysis. Given its scope, the ABCD Study protocol does not incorporate a process to achieve consensus for a final diagnostic profile for the youth. Hence, we considered information from the parent-report and child-report K-SADS-COMP separately. Although incorporating data from both reports is a strength, when there are large discrepancies between these reports, it is not always obvious which is more reliable, and this may depend on the specific diagnosis. For example, for internalizing psychopathology (eg, depressive disorders), child report may be a more reliable measure,^44^ while for ADHD, an externalizing psychopathology, parent report may be preferable.^45^ Additionally, recent work shows that the prevalence estimates of present ADHD in the baseline ABCD sample are reduced when requiring convergence of multiple informants or reports.^46^ Further, there are potential limitations due to K-SADS-COMP scores being based only on reported information and not the assessment of a trained clinician, which could mitigate concerns regarding the validity of certain diagnoses, such as bipolar disorder. Future research would benefit from a thorough and careful investigation into the prevalence and predictors of psychiatric diagnoses in the ABCD Study using information from multiple informants where possible. Additionally, reports of medication usage and other treatments are based on parent report and thus subject to potential biases, misunderstandings, and omissions.

With respect to limitations specific to the current project, we did not report on autism spectrum disorders and other neurodevelopmental disorders due to the limited reliability of the K-SADS for diagnosing these disorders. Moreover, we did not report on disruptive mood regulation disorder due to extremely low prevalence rates in the sample. Finally, in considering sociodemographic predictors of treatments, interaction effects were not assessed. Given the importance of considering intersectionality of identities,^47^ future work will benefit from investigating the potential interplay between factors as well as diagnostic comorbidity. Measures now being collected in the ABCD Study that were unavailable at baseline, such as insurance type, as well as other structural factors, such as perceived discrimination, should also be incorporated into future research. Despite these limitations, the present work is able to leverage many strengths of the large, population-based ABCD Study, and the numerous other neuroimaging, sociocultural, and biological measures collected in the study provide many future directions to better contextualize and explicate these findings.

In conclusion, prevalence rates of psychiatric disorders in the baseline ABCD sample are broadly similar to those expected based on prior prevalence studies in children and adolescents. Overall, psychiatric treatments align with expected patterns based on the psychopathology evident in the ABCD assessments. However, treatment patterns vary across sociodemographic categories, suggesting mental health disparities in how psychiatric treatments are offered and accessed in children living in the United States. The findings provide a foundation of understanding that will be important for guiding future research aimed at addressing current gaps in our pediatric mental health care system. Given that nearly half of children ages 4 to 18 with a mental health disorder do not receive treatment for their condition^3^ and that children’s mental health needs continue to increase^48^^,^^49^ with known mental health disparities in children and adolescents,^37^^,^^50^ informed policy is essential to ensuring equitable access to services for all.
