# Supplementary material for: Psychiatric Diagnoses and Treatment in Nine- to Ten-Year-Old Participants in the ABCD Study
Source: JAACAP Open. 2023 Mar 9;1(1):36–47. doi: 10.1016/j.jaacop.2023.03.001 (PMC10890826; doi:10.1016/j.jaacop.2023.03.001)
Supplement: Supplemental Data [file mmc1.docx]

**Supplemental Materials**

**Supplement 1**

*Additional Information on ABCD Sampling and Recruitment*

ABCD recruitment was based on the 21 pre-determined primary study sites. While the sites were intended to be representative of the United States, sites were selected based on their suitability in terms of neuroimaging equipment and relevant expertise needed to carry out the study protocol. As such, the site selection was not a random selection from all possible US sites, and further, sites were more likely to be in urban areas housing large research centers than in rural areas. Outside of this issue, though, the recruitment strategy was akin to a multi-stage probability sampling approach. Subjects were recruited primarily via schools. For each of the 21 study sites, defined catchment areas were created and schools within the catchment area were probabilistically sampled and eligible children were recruited from each school. The distribution of sociodemographic targets (racial and ethnic identities, age, gender, urbanicity, and socioeconomic status) within the catchment areas closely resembled the US distribution as a whole based on the American Community Survey (ACS) and data on 3rd and 4th graders maintained by the National Center for Education Statistics (NCES). There was, however, a slight intentional oversampling of particular racial and ethnic groups (Black/African-American, Other Race, and American Indian/Indigenous) to match study objectives and analytical aims. This was achieved primarily via oversampling schools meeting certain demographic proportions. Schools in rural and non-urban areas were also oversampled to address the aforementioned issue of rural under-representation in the study sites. In addition to school recruitment, a minority (less than 10%) of the sample was recruited via other approaches, such as through summer programming, referral from other participating subjects, and community outreach. The demographics of subjects recruited via alternative approaches were carefully monitored for any deviations from expectations that may indicate a sampling bias, and monitoring of the sample composition as a whole was performed throughout recruitment to ensure approximation of national targets. For more detailed information on the sampling and recruitment strategy, please see Garavan et al., 2018.

Twins were recruited in an alternative manner. Information from birth registries and twin registries maintained by the relevant study sites were used to initiate direct contact with the twins and parents (by using parental names and contact information from the registries to track parents and twins to their current residential addresses). As the twin registries maintained by the sites indicated that the overall racial and ethnic composition of twin births for the four study sites recruiting twins were disproportionately white relative to national measures, twins identifying as Hispanic/Latino/Latina, Asian, Native Hawaiian, Pacific Islander, Alaskan Native, American Indian, and Other Race were intentionally oversampled to more closely approximate national percentages. For more information on the twin recruitment strategy, please see Iacono et al., 2018.

**Table S1. Medications in each category reported in the ABCD sample at baseline**

| **Category** | **ATC code** | **Medication** |
| --- | --- | --- |
| *Stimulants* | N06B | methylphenidate, atomoxetine, dextroamphetamine, amphetamine, lisdexamfetamine, dexmethylphenidate, prescribed caffeine (*n*=1) |
| *Antidepressants* | N06A | escitalopram, fluoxetine, sertraline, mirtazapine, citalopram, fluvoxamine, imipramine, bupropion, amitriptyline, trazodone |
| *Antipsychotics* | N05A | risperidone, quetiapine, lurasidone, aripiprazole, clozapine, ziprasidone, lithium |
| *Anxiolytics* | N05B | hydroxyzine, buspirone, diazepam, alprazolam |
| *Hypnotics and Sedatives* | N05C | melatonin, scopolamine |

**Table S2. Prevalence of Clinically-Significant Symptom Reports According to KSADS-COMP and CBCL Reports in the ABCD Sample - Unweighted**

|  | **Parent Report** | **Child Report** |
| --- | --- | --- |
|  | **Percentage (%)** | **Percentage (%)** |
| **Prevalence of Psychiatric Disorders according to Symptom Reports on the KSADS-COMP** | | |
| **Depressive Disorders** | 6.2% | 5% |
| **Bipolar Disorder** | 6.8% | 9.2% |
| **Anxiety Disorders** | 33.1% | 1.3% |
| **Psychotic Disorders** | 0.6% | - |
| **Obsessive Compulsive Disorder (OCD)** | 9.3% | - |
| **Eating Disorders** | 0.9% | - |
| **Oppositional Defiant Disorder (ODD)** | 14% | - |
| **Conduct Disorder** | 3.2% | - |
| **Post-traumatic Stress Disorder (PTSD)** | 1.9% | - |
| **Substance Use Disorder** | 0.1% | - |
| **Attention-deficit Hyperactivity Disorder (ADHD)** | 18.4% | - |
| **Prevalence of Clinically-Significant CBCL Internalizing and Externalizing Total Scores** | | |
| **CBCL Internalizing *t*-score > 65** | 6.9% | - |
| **CBCL Externalizing *t*-score > 65** | 4.5% | - |

Note: Total *N* is 11,878. For the Diagnostic Clusters reported in subsequent tables, the “Depression,” “Anxiety,” and “Bipolar” clusters combine parent and child report of depressive disorders, anxiety disorders, and bipolar disorders, respectively, and the “Disruptive Behavior Disorders” cluster combines the Oppositional Defiant Disorder and Conduct Disorder diagnoses. Note that for the “Anxiety” disorders cluster only, the specific disorders included in parent and child report differ: parent report evaluates symptoms of social anxiety disorder, selective mutism, panic disorder, agoraphobia, separation anxiety disorder, specific phobia, and generalized anxiety disorder, while child report only evaluates symptoms of social anxiety disorder and generalized anxiety disorder. See Table S6 for more information. CBCL = Child Behavior Checklist; KSADS-COMP = Computerized Kiddie Schedule for Affective Disorders and Schizophrenia.

**Table S3. Prevalence of Treatment – Unweighted**

| Intervention | Sample % |
| --- | --- |
| Medications^a^ |  |
| At Least One Class of Medications | 9.2% |
| Stimulants | 7.8% |
| Antidepressants | 1.8% |
| Antipsychotics | 0.6% |
| Sedatives | 0.2% |
| Anxiolytics | 0.2% |
| Other Interventions for Mental Health |  |
| At Least One Intervention | 14.9% |
| Outpatient^b^ | 8.7% |
| Partial Hospitalization | 0.2% |
| Inpatient | 0.5% |
| Psychotherapy | 4.6% |
| Other | 2.9% |

Note: Total *N* is 11,878.

^a^Medication classes defined using the Anatomical Therapeutic Chemical Classification system.

^b^Note that categorizations of treatment levels were based on parental discretion and not mutually exclusive, and “outpatient” treatment in particular is a level of care which may potentially encompass several types of treatment, including medication visits and psychotherapy.

**Table S4. Clinical Predictors of Interventions: Adjusted Odds Ratios for Medication and Treatment by KSADS-COMP Diagnosis and CBCL Internalizing and Externalizing Scores - Unweighted**

|  | **At Least One Medication** | **Stimulants** | **Antidep- ressants** | **Anti- psychotics** | **Sedatives** | **Anxiolytics** | **At Least One Other Intervention** | **Outpatient**^a^ | **Partial Hospitaliz-ation** | **Inpatient** | **Psycho- therapy** | **Other** |
| --- | --- | --- | --- | --- | --- | --- | --- | --- | --- | --- | --- | --- |
| **Intercept** | 0.03*** | 0.03*** | 0*** | 0*** | 0*** | 0*** | 0.05*** | 0.03*** | 0*** | 0*** | 0.02*** | 0.01*** |
| **CBCL Internalizing (raw scores)** | 1.01 | 0.99 | 1.06*** | 0.99 | 1.08* | 1.05 | 1.06*** | 1.04*** | 1.02 | 0.98 | 1.06*** | 1.03* |
| **CBCL Externalizing (raw scores)** | 1.05*** | 1.05*** | 1.03* | 1.12*** | 1 | 1.08 | 1.05*** | 1.06*** | 1.09* | 1.1*** | 1 | 1.04*** |
| **Depression** | 1.17 | 0.93 | 2.06*** | 1.79 | 1.59 | 1.53 | 1.55*** | 1.48*** | 1.18 | 1.85 | 1.38* | 1.16 |
| **Bipolar** | 1.26** | 1.15 | 1.64** | 1.47 | 2.28 | 0.99 | 1.52*** | 1.63*** | 0.8 | 0.87 | 1.46*** | 1.25 |
| **Anxiety** | 1.18 | 1.27* | 0.88 | 1.77 | 1.22 | 2.54 | 1.1 | 0.99 | 2.02 | 2.39* | 1.19 | 1.1 |
| **Psychosis (Parent report)** | 1.48 | 1.07 | 1.08 | 2.45 | 4.1 | 0 | 1.16 | 1.42 | 1.06 | 1.39 | 1.12 | 0.56 |
| **OCD (Parent report)** | 1.23 | 1.23 | 1.46* | 1.4 | 0.64 | 0.88 | 1.17 | 1 | 1.59 | 0.77 | 1.19 | 1.43* |
| **Eating Disorder (Parent report)** | 0.85 | 0.65 | 1.54 | 1.07 | 2.68 | 0 | 1.27 | 0.98 | 2.7 | 2.38 | 1.49 | 1.59 |
| **ODD/CD (Parent report)** | 1.31** | 1.27* | 1.84** | 1.69 | 1.35 | 0.74 | 1.98*** | 1.94*** | 1.96 | 2.25 | 1.85*** | 1.34 |
| **PTSD (Parent report)** | 1.01 | 1.01 | 0.92 | 0.82 | 0.26 | 0.74 | 2.29*** | 2.71*** | 0.67 | 2.38 | 1.04 | 0.84 |
| **ADHD (Parent report)** | 5.6*** | 7.2*** | 2.04*** | 1.69 | 2.48 | 1.85 | 1.79*** | 1.68*** | 2.09 | 1.77 | 1.79*** | 1.42* |

Note: Note that adjusted odds ratios of 0 in the table are rounded to zero and do not represent true zero values, but rather reflect that there were no observed instances of the combination of treatment type and clinical diagnosis in the sample. ADHD = Attention-deficit/hyperactivity disorder; CBCL = Child Behavior Checklist; KSADS-COMP = Computerized Kiddie Schedule for Affective Disorders and Schizophrenia; OCD = Obsessive-compulsive disorder; ODD/CD = Oppositional defiant disorder/Conduct disorder; PTSD = Post-traumatic stress disorder.

^a^Note that categorizations of treatment levels were based on parental discretion and not mutually exclusive, and “outpatient” treatment in particular is a level of care which may potentially encompass several types of treatment, including medication visits and psychotherapy.

**p* < .05; ***p* < .01; ****p* <.001; *p*-values adjusted for multiple comparisons using Benjamini-Hochberg method.

**Table S5. Sociodemographic Predictors of Interventions: Adjusted Odds Ratios - Unweighted**

|  | **At Least One Medication** | **Stimulants** | **Antidep- ressants** | **Anti- psychotics** | **Sedatives** | **Anxiolytics** | **At Least One Other Intervention** | **Outpatient**^a^ | **Partial Hospitaliz- ation** | **Inpatient** | **Psycho- therapy** | **Other** |
| --- | --- | --- | --- | --- | --- | --- | --- | --- | --- | --- | --- | --- |
| **(Intercept)** | 0.04*** | 0.04*** | 0*** | 0*** | 0*** | 0*** | 0.11*** | 0.08*** | 0.01*** | 0*** | 0.01*** | 0.01*** |
| **Sex at Birth: Male** | 2.39*** | 2.71*** | 1.48* | 3.29** | 1.62 | 2.12 | 1.43*** | 1.43*** | 1.4 | 2.28* | 1.26 | 1.51** |
| **Household Income: >=50K & <100K** | 0.65*** | 0.68*** | 0.69 | 0.54 | 1.1 | 0.17* | 0.58*** | 0.55*** | 0.3 | 0.43 | 0.74 | 0.71 |
| **Household Income: >=100K** | 0.58*** | 0.59*** | 0.69 | 0.32** | 0.73 | 0.03** | 0.53*** | 0.43*** | 0 | 0.19** | 0.92 | 0.63* |
| **Parental Education: Any College** | 1.32* | 1.19 | 4.85** | 1.58 | 1.13 | 4.24 | 1.61*** | 1.32* | 0.53 | 1.36 | 2.36** | 1.85* |
| **Asian** | 0.71 | 0.75 | 0.59 | 0.34 | 1.17 | 1.1 | 0.75* | 0.81 | 0 | 0 | 0.68 | 1.02 |
| **Black** | 1.4** | 1.44* | 1.39 | 1.69 | 0.45 | 0.94 | 1.06 | 1.11 | 1.06 | 2.38 | 0.99 | 1.2 |
| **Indigenous**^b^ | 1.23 | 1.25 | 0.63 | 1.54 | 3.38 | 3.91 | 1.09 | 1.23 | 1.47 | 0.92 | 0.58 | 0.96 |
| **White** | 1.6*** | 1.59** | 2.23* | 1.66 | 0.8 | 1.68 | 1.71*** | 1.7*** | 1.08 | 1.61 | 2.01** | 1.4 |
| **Other Race** | 0.9 | 0.84 | 1.72 | 1.36 | 0 | 0 | 0.95 | 0.8 | 1.28 | 0 | 0.77 | 1.76 |
| **Hispanic/Latino/Latina** | 0.72** | 0.82 | 0.36** | 0.58 | 0.46 | 0.2 | 0.58*** | 0.49*** | 0.28 | 0.3 | 0.87 | 0.7 |
| **Urbanicity: Urban Cluster** | 1.58** | 1.53* | 1.38 | 2.96 | 1.23 | 1.26 | 0.95 | 1.02 | 1.62 | 1.59 | 0.59 | 1.44 |
| **Urbanicity: Rural** | 1.22 | 1.3 | 0.8 | 1.61 | 2.92 | 0 | 0.77* | 0.86 | 0 | 0.53 | 0.7 | 0.59 |

Note: Note that adjusted odds ratios of 0 in the table are rounded to zero and do not represent true zero values, but rather reflect that there were no observed instances of the combination of treatment type and demographic characteristic in the sample.

^a^Note that categorizations of treatment levels were based on parental discretion and not mutually exclusive, and “outpatient” treatment in particular is a level of care which may potentially encompass several types of treatment, including medication visits and psychotherapy. ^b^American Indian, Alaska Native, Native Hawaiian, or Pacific Islander

**p* < .05; ***p* < .01; ****p* <.001; *p*-values adjusted for multiple comparisons using Benjamini-Hochberg method.

**Table S6. Prevalence of all specific psychiatric disorders included in larger diagnostic classes in the ABCD sample according to the KSADS-COMP - Weighted and Unweighted Percentages**

| **Disorder** | **Weighted Percentage** | **Unweighted Percentage** |
| --- | --- | --- |
| **Depression** | | |
| *Parent Report* | | |
| Persistent Depressive Disorder (Dysthymia) - Present | 0% | 0% |
| Persistent Depressive Disorder (Dysthymia) - In Partial Remission | 0% | 0% |
| Major Depressive Disorder - Present | 0.24% | 0.19% |
| Major Depressive Disorder - In Partial Remission | 0.16% | 0.16% |
| Unspecified Depressive Disorder - Current | 0.1% | 0.12% |
| Persistent Depressive Disorder (Dysthymia) - Past | 0.18% | 0.14% |
| Major Depressive Disorder - Past | 2.66% | 2.45% |
| Unspecified Depressive Disorder - Past | 3.61% | 3.52% |
| *Child Report* | | |
| Persistent Depressive Disorder (Dysthymia) - Present | 0% | 0% |
| Persistent Depressive Disorder (Dysthymia) - In Partial Remission | 0% | 0% |
| Major Depressive Disorder - Present | 0.63% | 0.56% |
| Major Depressive Disorder - In Partial Remission | 0.22% | 0.23% |
| Unspecified Depressive Disorder - Current | 0.25% | 0.24% |
| Persistent Depressive Disorder (Dysthymia) - Past | 0.08% | 0.06% |
| Major Depressive Disorder - Past | 2.36% | 2.15% |
| Unspecified Depressive Disorder - Past | 2.3% | 2.18% |
| **Bipolar** | | |
| *Parent Report* | | |
| Bipolar II, Most Recent Past Hypomanic | 1.24% | 1.09% |
| Bipolar II, Currently Hypomanic | 0% | 0% |
| Bipolar II, Currently Depressed | 0.05% | 0.06% |
| Bipolar I, Current Episode Depressed | 0.11% | 0.1% |
| Bipolar I, Currently Hypomanic | 0.02% | 0.01% |
| Bipolar I, Current Episode Manic | 0.16% | 0.15% |
| Bipolar I, Most Recent Past Episode Manic | 2.72% | 2.56% |
| Bipolar I, Most Recent Past Episode Depressed | 0.18% | 0.18% |
| Unspecified Bipolar and Related Disorder - Current | 0.38% | 0.35% |
| Unspecified Bipolar and Related Disorder - Past | 3.57% | 3.3% |
| *Child Report* | | |
| Bipolar II, Most Recent Past Hypomanic | 1.19% | 1.01% |
| Bipolar II, Currently Hypomanic | 0.08% | 0.07% |
| Bipolar II, Currently Depressed | 0.21% | 0.19% |
| Bipolar I, Current Episode Depressed | 0.22% | 0.21% |
| Bipolar I, Currently Hypomanic | 0.08% | 0.05% |
| Bipolar I, Current Episode Manic | 0.23% | 0.23% |
| Bipolar I, Most Recent Past Episode Manic | 3.12% | 2.98% |
| Bipolar I, Most Recent Past Episode Depressed | 0.35% | 0.29% |
| Unspecified Bipolar and Related Disorder - Current | 0.68% | 0.63% |
| Unspecified Bipolar and Related Disorder - Past | 5.17% | 5.09% |
| **Anxiety** | | |
| *Parent Report* | | |
| Social Anxiety Disorder - Present | 0.99% | 0.93% |
| Selective Mutism - Present | 0% | 0% |
| Panic Disorder - Present | 0.04% | 0.04% |
| Agoraphobia - Present | 0.14% | 0.12% |
| Separation Anxiety Disorder - Present | 0.43% | 0.4% |
| Specific Phobia - Present | 9.16% | 8.73% |
| Generalized Anxiety Disorder - Present | 1.09% | 1.09% |
| Social Anxiety Disorder - Past | 4.23% | 4.26% |
| Selective Mutism - Past | 0% | 0% |
| Panic Disorder - Past | 0.24% | 0.25% |
| Agoraphobia - Past | 0.45% | 0.4% |
| Separation Anxiety Disorder - Past | 9.06% | 8.79% |
| Specific Phobia - Past | 23.2% | 22.71% |
| Generalized Anxiety Disorder - Past | 3.85% | 3.7% |
| *Child Report* | | |
| Social Anxiety Disorder - Present | 0.46% | 0.4% |
| Generalized Anxiety Disorder - Present | 0.34% | 0.29% |
| Social Anxiety Disorder - Past | 0.48% | 0.5% |
| Generalized Anxiety Disorder - Past | 0.49% | 0.47% |
| **DMDD** | | |
| *Parent Report* | | |
| Disruptive Mood Regulation Disorder (DMDD) - Current | 0.07% | 0.07% |
| *Child Report* | | |
| Disruptive Mood Regulation Disorder (DMDD) - Current | 0.12% | 0.12% |
| **Psychosis (Parent Report)** | | |
| Unspecified Schizophrenia Spectrum and Other Psychotic Disorder - Current | 0.31% | 0.28% |
| Unspecified Schizophrenia Spectrum and Other Psychotic Disorder - Past | 0.51% | 0.5% |
| **OCD (Parent Report)** | | |
| Obsessive-Compulsive Disorder - Present | 7.92% | 7.57% |
| Obsessive-Compulsive Disorder - Past | 4.23% | 4.21% |
| **Eating Disorder (Parent Report)** | | |
| Bulimia Nervosa - Present | 0.05% | 0.05% |
| Bulimia Nervosa - Partial Remission | 0% | 0% |
| Anorexia Nervosa, Binge eating/purging subtype - Present | 0% | 0% |
| Anorexia Nervosa, Binge eating/purging subtype - Partial Remission | 0% | 0% |
| Anorexia Nervosa, Restricting subtype - Present | 0.01% | 0.01% |
| Anorexia Nervosa, Restricting subtype - Partial Remission | 0.03% | 0.04% |
| Binge-Eating Disorder - Current | 0.66% | 0.62% |
| Binge-Eating Disorder - In Partial Remission | 0% | 0% |
| Bulimia Nervosa - Past | 0.01% | 0.01% |
| Anorexia Nervosa, Restricting subtype - Past | 0.05% | 0.04% |
| Anorexia Nervosa, Binge eating/purging subtype - Past | 0% | 0% |
| Binge-Eating Disorder - Past | 0.34% | 0.35% |
| **Disruptive Behavior Disorders (Parent Report)** | | |
| Oppositional Defiant Disorder - Present | 5.46% | 5.54% |
| Oppositional Defiant Disorder - Past | 9.14% | 9.01% |
| Conduct Disorder, Childhood Onset - Present | 2.87% | 2.8% |
| Conduct Disorder, Adolescent Onset - Present | 0.4% | 0.35% |
| Conduct Disorder, Childhood Onset - Past | 1.04% | 0.99% |
| Conduct Disorder, Adolescent Onset - Past | 0.21% | 0.19% |
| **PTSD (Parent Report)** | | |
| Post-Traumatic Stress Disorder - Present | 0.12% | 0.11% |
| Post-Traumatic Stress Disorder - Past | 2.09% | 1.9% |
| **Substance Use Disorder (Parent Report)** | | |
| Alcohol Use Disorder - Present | 0% | 0% |
| Stimulant Use Disorder, Cocaine - Present | 0% | 0% |
| Stimulant Use Disorder, Amphetamine-type substance - Present | 0.08% | 0.08% |
| Inhalant Use Disorder - Present | 0% | 0% |
| Phencyclidine (PCP) Use Disorder - Present | 0% | 0% |
| Opioid Use Disorder - Present | 0% | 0% |
| Other Hallucinogen Disorder - Present | 0% | 0% |
| Sedative, Hypnotic or Anxiolytic Use Disorder - Present | 0% | 0% |
| Cannabis Use Disorder - Present | 0% | 0% |
| Alcohol Use Disorder - Past | 0% | 0% |
| Stimulant Use Disorder, Cocaine - Past | 0% | 0% |
| Stimulant Use Disorder, Amphetamine-type substance - Past | 0.01% | 0.01% |
| Inhalant Use Disorder - Past | 0% | 0% |
| Phencyclidine (PCP) Use Disorder - Past | 0% | 0% |
| Opioid Use Disorder - Past | 0% | 0% |
| Other Hallucinogen Disorder - Past | 0% | 0% |
| Sedative, Hypnotic or Anxiolytic Use Disorder - Past | 0.01% | 0.01% |
| Cannabis Use Disorder - Past | 0% | 0% |
| **ADHD (Parent Report)** | | |
| Attention-Deficit/Hyperactivity Disorder - Present | 9% | 9.13% |
| Attention-Deficit/Hyperactivity Disorder - Past | 10.99% | 10.48% |

Note: Total *N* is 11,878. Weighted percentages are weighted to match American Community Survey (ACS) demographic proportions.

**Table S7. Prevalence of interventions for subsets of the ABCD sample with different classes of KSADS-COMP-reported psychiatric diagnoses - Weighted**

| **Diagnostic Clusters^a^** | **Subset w/ Anxiety** | **Subset w/ Bipolar** | **Subset w/ Depression** | **Subset w/ ADHD (Parent report)** | **Subset w/ Disruptive Behavior Disorders (Parent report)** | **Subset w/ OCD (Parent report)** | **Subset w/ PTSD (Parent report)** | **Subset w/ Eating Disorder (Parent report)** | **Subset w/ Psychosis (Parent report)** | **Subset w/ Substance Use Disorder (Parent report)** |
| --- | --- | --- | --- | --- | --- | --- | --- | --- | --- | --- |
| **Medications^b^** | | | | | | | | | | |
| **At Least One Class of Medications** | 15.3% | 18% | 18.3% | 30.9% | 26% | 21.4% | 28.8% | 20.6% | 36.7% | 63.5% |
| **Stimulants** | 12.6% | 15.5% | 13.7% | 27.8% | 22.1% | 17.9% | 22.7% | 15% | 28.5% | 63.5% |
| **Antidepressants** | 3.7% | 3.8% | 5.8% | 5.7% | 6.3% | 5.8% | 8.5% | 9% | 8.1% | 0% |
| **Antipsychotics** | 1.2% | 2.1% | 2.4% | 2.3% | 2.9% | 2.3% | 2.5% | 3.5% | 7.3% | 0% |
| **Sedatives** | 0.5% | 0.5% | 0.7% | 0.7% | 0.7% | 0.5% | 0.3% | 2.8% | 2.4% | 7.2% |
| **Anxiolytics** | 0.3% | 0.7% | 0.7% | 0.5% | 0.4% | 0.5% | 0.3% | 0% | 0% | 0% |
| **Other Interventions for Mental Health** | | | | | | | | | | |
| **At Least One Intervention** | 25.7% | 26.5% | 32.1% | 35.1% | 41.9% | 33.2% | 61.8% | 46% | 50.1% | 55.4% |
| **Outpatient** | 15.7% | 16% | 0.5% | 22.1% | 27.5% | 20% | 47.1% | 28.9% | 37.1% | 36% |
| **Partial Hospitalization** | 0.3% | 0.5% | 2.1% | 0.7% | 0.8% | 0.7% | 0.8% | 1.7% | 2% | 0% |
| **Inpatient** | 1% | 1.9% | 10.9% | 2% | 2.6% | 1.3% | 6.2% | 7.2% | 6.4% | 19.3% |
| **Psychotherapy** | 8.4% | 8.7% | 4.5% | 11.3% | 12.7% | 11.1% | 18.3% | 17.7% | 16.7% | 18.9% |
| **Other** | 4.3% | 4.7% | 0.5% | 5.6% | 6.4% | 6.4% | 6.9% | 8.4% | 5.5% | 0% |

Note: Total *N* is 11,878. Weighted percentages are weighted to match American Community Survey (ACS) demographic proportions.

**^a^**Diagnosis clusters are defined by the presence of diagnosis per Parent KSADS and/or (if available for a given diagnosis) child KSADS, combining current, partial remission and/or past diagnoses. Depression cluster include major depressive disorder, unspecified depression, persistent depressive disorder; Bipolar cluster includes bipolar disorder subtypes 1,2, and unspecified; Psychosis refers to Unspecified Schizophrenia Spectrum and Other Psychotic Disorder; Anxiety cluster includes generalized anxiety disorder, social phobia, specific phobia, separation anxiety disorder, panic disorder, unspecified anxiety disorder, selective mutism, agoraphobia; Disruptive Behavior Disorders cluster includes oppositional defiant disorder and conduct disorder; Eating Disorder cluster includes anorexia nervosa, bulimia nervosa, and binge eating disorder, and “Other Specified Feeding or Eating Disorder Bulimia Nervosa current does not meet full criteria”.

**^b^**Medication classes defined using the Anatomical Therapeutic Chemical Classification system.

**Table S8. Prevalence of interventions for subsets of the ABCD sample with different classes of KSADS-COMP-reported psychiatric diagnoses- Unweighted**

| **Diagnostic Clusters^a^** | **Subset w/ Anxiety** | **Subset w/ Bipolar** | **Subset w/ Depression** | **Subset w/ ADHD (Parent report)** | **Subset w/ Disruptive Behavior Disorders (Parent report)** | **Subset w/ OCD (Parent report)** | **Subset w/ PTSD (Parent report)** | **Subset w/ Eating Disorder (Parent report)** | **Subset w/ Psychosis (Parent report)** | **Subset w/ Substance Use Disorder (Parent report)** |
| --- | --- | --- | --- | --- | --- | --- | --- | --- | --- | --- |
| **Medications^b^** | | | | | | | | | | |
| **At Least One Class of Medications** | 14.4% | 16.9% | 17.5% | 30.4% | 25.3% | 21% | 28.6% | 21.9% | 38.6% | 60% |
| **Stimulants** | 11.7% | 14.7% | 12.8% | 27.5% | 21.5% | 17.2% | 22.5% | 14.3% | 27.1% | 60% |
| **Antidepressants** | 3.6% | 3.3% | 6.1% | 5.5% | 6.2% | 6% | 8.7% | 10.5% | 10% | 0% |
| **Antipsychotics** | 1.2% | 1.9% | 2.1% | 2.2% | 2.7% | 2.3% | 3.5% | 3.8% | 8.6% | 0% |
| **Sedatives** | 0.4% | 0.4% | 0.6% | 0.6% | 0.6% | 0.5% | 0.4% | 1.9% | 2.9% | 10% |
| **Anxiolytics** | 0.3% | 0.6% | 0.5% | 0.5% | 0.4% | 0.5% | 0.4% | 0% | 0% | 0% |
| **Other Interventions for Mental Health** | | | | | | | | | | |
| **At Least One Intervention** | 25% | 25.3% | 32.3% | 34.5% | 40.7% | 33% | 60.6% | 44.8% | 52.9% | 50% |
| **Outpatient** | 15.4% | 15.4% | 20.4% | 21.8% | 26.7% | 19.8% | 47.6% | 27.6% | 40% | 30% |
| **Partial Hospitalization** | 0.3% | 0.6% | 0.5% | 0.6% | 0.7% | 0.6% | 0.9% | 1.9% | 1.4% | 0% |
| **Inpatient** | 0.9% | 1.8% | 1.8% | 1.8% | 2.3% | 1.3% | 5.2% | 5.7% | 4.3% | 20% |
| **Psychotherapy** | 8% | 8.3% | 10.3% | 11% | 12.3% | 11.1% | 16.5% | 18.1% | 18.6% | 10% |
| **Other** | 4.3% | 4.7% | 5.3% | 5.9% | 6.7% | 6.6% | 7.4% | 10.5% | 5.7% | 0% |

Note: Total *N* is 11,878.

**^a^**Diagnosis clusters are defined by the presence of diagnosis per Parent KSADS and/or (if available for a given diagnosis) child KSADS, combining current, partial remission and/or past diagnoses. Depression cluster include major depressive disorder, unspecified depression, persistent depressive disorder; Bipolar cluster includes bipolar disorder subtypes 1,2, and unspecified; Psychosis refers to Unspecified Schizophrenia Spectrum and Other Psychotic Disorder; Anxiety cluster includes generalized anxiety disorder, social phobia, specific phobia, separation anxiety disorder, panic disorder, unspecified anxiety disorder, selective mutism, agoraphobia; Disruptive Behavior Disorders cluster includes oppositional defiant disorder and conduct disorder; Eating Disorder cluster includes anorexia nervosa, bulimia nervosa, and binge eating disorder, and “Other Specified Feeding or Eating Disorder Bulimia Nervosa current does not meet full criteria”.

**^b^**Medication classes defined using the Anatomical Therapeutic Chemical Classification system.

**Table S9. Prevalence of classes of psychiatric diagnoses according to the KSADS-COMP based on sociodemographic variables in sample - Weighted Counts and Percentages**

|  |  | **Depression** | **Bipolar** | **Anxiety** | **Psychosis (Parent report)** | **OCD (Parent report)** | **Eating Disorder (Parent report)** | **ODD/CD (Parent report)** | **PTSD (Parent report)** | **Substance Use Disorder (Parent report)** | **ADHD (Parent report)** |
| --- | --- | --- | --- | --- | --- | --- | --- | --- | --- | --- | --- |
| **Sex at Birth** | Female | 10.5% | 14.1% | 34.4% | 0.5% | 7.9% | 0.8% | 11.8% | 2% | 0% | 13.4% |
|  | Male | 12.2% | 17.7% | 34.2% | 0.8% | 11.1% | 1.1% | 18.5% | 2.3% | 0.2% | 23.9% |
| **Household Income** | <50K | 15.3% | 20.2% | 37.5% | 0.9% | 12.2% | 1.5% | 16.5% | 3.6% | 0.1% | 21.1% |
|  | >=50K & <100K | 9.7% | 14.3% | 34.2% | 0.4% | 8.3% | 0.7% | 15.3% | 1.4% | 0.1% | 19.1% |
|  | >=100K | 8% | 11.2% | 31.5% | 0.4% | 6.8% | 0.5% | 14.1% | 1.1% | 0.1% | 16.2% |
| **Urbanicity** | Urbanized Area | 11.4% | 15.8% | 33.9% | 0.6% | 9.2% | 0.8% | 14.8% | 2.2% | 0.1% | 18.3% |
|  | Urban Cluster | 11.8% | 18.1% | 36.1% | 1.5% | 12.8% | 1.3% | 19.3% | 2.9% | 0% | 20.3% |
|  | Rural | 11.4% | 17.4% | 37.1% | 0.2% | 10.3% | 1.5% | 17.6% | 1.5% | 0% | 22.5% |
| **Parental Education** | No College | 13.9% | 20.2% | 31.7% | 0.8% | 12.3% | 0.9% | 13.8% | 2.6% | 0% | 15.9% |
|  | Any College | 10.8% | 15% | 34.9% | 0.6% | 9% | 0.9% | 15.5% | 2% | 0.1% | 19.4% |
| **Race** | Asian | 5.7% | 12.3% | 28.4% | 0% | 6.9% | 0.5% | 8.3% | 1.5% | 0.1% | 11.8% |
|  | Black | 14.5% | 22.7% | 33.7% | 1.3% | 12% | 1.5% | 16.1% | 3.2% | 0.2% | 20.8% |
|  | Native American/Alaskan Native | 13.1% | 26.1% | 35.3% | 0.4% | 11.6% | 1% | 15% | 3.3% | 0.3% | 18.5% |
|  | Pacific Islander/Native Hawaiian | 18.4% | 13.7% | 29.3% | 0% | 7.1% | 1.1% | 12.9% | 2.9% | 0% | 13.9% |
|  | White | 11.1% | 14.5% | 35.5% | 0.5% | 9.5% | 0.9% | 16.2% | 2% | 0.1% | 19.5% |
|  | Other Race | 13.2% | 17.8% | 34.8% | 1.3% | 9.8% | 1.9% | 14.5% | 3.6% | 0.2% | 16.2% |
| **Ethnicity** | Hispanic/Latino/Latina | 12% | 15.9% | 32.9% | 0.7% | 9.8% | 1.3% | 11.3% | 2% | 0.2% | 16.8% |

Note: Total *N* is 11,878. Percentages are weighted to match American Community Survey (ACS) demographic proportions.

**Table S10. Prevalence of classes of psychiatric diagnoses according to the KSADS-COMP based on sociodemographic variables in sample - Unweighted Counts and Percentages**

|  |  | **Depression** | **Bipolar** | **Anxiety** | **Psychosis (Parent report)** | **OCD (Parent report)** | **Eating Disorder (Parent report)** | **ODD/CD (Parent report)** | **PTSD (Parent report)** | **Substance Use Disorder (Parent report)** | **ADHD (Parent report)** |
| --- | --- | --- | --- | --- | --- | --- | --- | --- | --- | --- | --- |
| **Sex at Birth** | Female | 10% | 13.3% | 33.9% | 0.4% | 7.8% | 0.8% | 11.7% | 1.8% | 0% | 13.1% |
|  | Male | 11.4% | 16.8% | 33.5% | 0.7% | 10.6% | 1% | 18.3% | 2.1% | 0.1% | 23.3% |
| **Household Income** | <50K | 15.5% | 20.6% | 37.1% | 1% | 12.7% | 1.6% | 16.4% | 3.7% | 0.2% | 20.8% |
|  | >=50K & <100K | 9.8% | 13.9% | 34% | 0.3% | 8% | 0.7% | 15.4% | 1.4% | 0% | 19.2% |
|  | >=100K | 7.9% | 11.5% | 31.6% | 0.5% | 7.1% | 0.6% | 14.2% | 1.1% | 0.1% | 16.5% |
| **Urbanicity** | Urbanized Area | 10.8% | 15% | 33.4% | 0.6% | 9.2% | 0.8% | 14.9% | 2% | 0.1% | 18.2% |
|  | Urban Cluster | 9.9% | 16.7% | 33.3% | 0.8% | 10.2% | 1.1% | 16.7% | 2.4% | 0% | 18.8% |
|  | Rural | 10.6% | 16.3% | 35.5% | 0.1% | 9.6% | 1.2% | 17.3% | 1.6% | 0% | 21% |
| **Parental Education** | No College | 14% | 20.2% | 32.3% | 0.9% | 12.5% | 1% | 13.8% | 2.5% | 0.1% | 15.8% |
|  | Any College | 10.2% | 14.3% | 33.9% | 0.5% | 8.7% | 0.9% | 15.4% | 1.8% | 0.1% | 18.9% |
| **Race** | Asian | 6.1% | 11.2% | 31.1% | 0% | 6.4% | 0.4% | 10.9% | 2% | 0.1% | 14.9% |
|  | Black | 14.5% | 22.4% | 33.8% | 1.1% | 11.6% | 1.4% | 16.3% | 3.1% | 0.2% | 21.3% |
|  | Native American/Alaskan Native | 15.4% | 24.1% | 37.1% | 0.5% | 12.9% | 1.2% | 17.8% | 4.1% | 0.2% | 24.6% |
|  | Pacific Islander/Native Hawaiian | 13.5% | 16.2% | 32.4% | 0% | 9.5% | 1.4% | 14.9% | 2.7% | 0% | 20.3% |
|  | White | 10.1% | 13.2% | 34.5% | 0.5% | 9% | 0.8% | 16% | 1.8% | 0.1% | 18.7% |
|  | Other Race | 13.1% | 17.2% | 33.9% | 1% | 9.9% | 1.9% | 13.1% | 2.8% | 0.2% | 15.1% |
| **Ethnicity** | Hispanic/Latino/Latina | 11.4% | 15.5% | 32.6% | 0.7% | 9.1% | 1.2% | 11.2% | 1.9% | 0.2% | 16.5% |

Note: Total *N* is 11,878.

**Table S11 - Sociodemographic predictors psychiatric diagnoses according to the KSADS-COMP – Adjusted Odds Ratios, Weighted**

|  | **Depression** | **Bipolar** | **Anxiety** | **Psychosis (Parent report)** | **OCD (Parent report)** | **Eating Disorder (Parent report)** | **ODD/CD (Parent report)** | **PTSD (Parent report)** | **ADHD (Parent report)** |
| --- | --- | --- | --- | --- | --- | --- | --- | --- | --- |
| **(Intercept)** | 0.13*** | 0.22*** | 0.42*** | 0*** | 0.08*** | 0*** | 0.1*** | 0.02*** | 0.1*** |
| **Sex at Birth: Male** | 1.2 | 1.36** | 0.96 | 1.75 | 1.51*** | 1.32 | 1.63*** | 1.2 | 2.02*** |
| **Household Income: >=50K & <100K** | 0.63** | 0.73* | 0.77* | 0.6 | 0.66* | 0.36* | 0.8 | 0.34* | 0.76* |
| **Household Income: >=100K** | 0.49*** | 0.57** | 0.65** | 0.72 | 0.54** | 0.28* | 0.69* | 0.24** | 0.59** |
| **Parental Education: Any College** | 0.9 | 0.94 | 1.23 | 1.32 | 0.88 | 1.58 | 1 | 1.23 | 1.48* |
| **Asian** | 0.55 | 0.88 | 0.89 | 0*** | 0.91 | 1.1 | 0.68* | 1.28 | 0.75 |
| **Black** | 1.36 | 1.3 | 1.09 | 2.62 | 1.35* | 1.79 | 1.37 | 1.72 | 1.4* |
| **Alaskan Native/American Indian/Native Hawaiian/Pacific Islander** | 1.28 | 1.61 | 1.06 | 0.64 | 1.14 | 0.93 | 1.12 | 1.23 | 0.98 |
| **White** | 1.39 | 0.87 | 1.46* | 1.21 | 1.38 | 2.24 | 1.92** | 2.02* | 1.52** |
| **Other Race** | 1.45 | 1.13 | 1.43* | 2.22 | 1.27 | 3.34 | 1.85* | 2.73* | 1.27 |
| **Hispanic/Latino/Latina** | 0.87 | 0.88 | 0.82* | 0.89 | 0.97 | 1.12 | 0.52** | 0.53 | 0.8 |
| **Urbanicity: Urban Cluster** | 0.97 | 1.31 | 0.98 | 2.75 | 1.49 | 1.41 | 1.12 | 1.19 | 1.02 |
| **Urbanicity: Rural** | 1.1 | 1.26 | 1.07 | 0.38 | 1.29** | 2.3 | 1.05 | 0.75 | 1.28* |

Note: Results are weighted to match American Community Survey (ACS) demographic proportions.

**p* < .05; ***p* < .01; ****p* <.001; *p*-values adjusted for multiple comparisons using Benjamini-Hochberg method.

**Table S12 - Sociodemographic predictors of classes of psychiatric diagnoses according to the KSADS-COMP – Adjusted Odds Ratios, Unweighted**

|  | **Depression** | **Bipolar** | **Anxiety** | **Psychosis (Parent report)** | **OCD (Parent report)** | **Eating Disorder (Parent report)** | **ODD/CD (Parent report)** | **PTSD (Parent report)** | **ADHD (Parent report)** |
| --- | --- | --- | --- | --- | --- | --- | --- | --- | --- |
| **(Intercept)** | 0.13*** | 0.2*** | 0.44*** | 0*** | 0.1*** | 0*** | 0.1*** | 0.02*** | 0.11*** |
| **Sex at Birth: Male** | 1.16* | 1.36*** | 0.96 | 1.9 | 1.44*** | 1.18 | 1.62*** | 1.23 | 1.98*** |
| **Household Income: >=50K & <100K** | 0.64*** | 0.74*** | 0.78*** | 0.44 | 0.6*** | 0.38** | 0.83* | 0.35*** | 0.8** |
| **Household Income: >=100K** | 0.5*** | 0.63*** | 0.66*** | 0.68 | 0.54*** | 0.35** | 0.71*** | 0.26*** | 0.65*** |
| **Parental Education: Any College** | 0.88 | 0.96 | 1.15 | 1.03 | 0.87 | 1.47 | 1 | 1.25 | 1.47*** |
| **Asian** | 0.59** | 0.77 | 0.99 | 0 | 0.83 | 0.68 | 0.76 | 1.5 | 0.84 |
| **Black** | 1.44** | 1.44*** | 1.11 | 2.38 | 1.29 | 1.73 | 1.38** | 1.72* | 1.36** |
| **Alaskan Native/American Indian/Native Hawaiian/Pacific Islander** | 1.42* | 1.57*** | 1.05 | 0.76 | 1.37 | 1.28 | 1.19 | 1.77 | 1.3* |
| **White** | 1.38** | 0.83 | 1.44*** | 1.43 | 1.35* | 1.90 | 1.83*** | 1.65 | 1.32** |
| **Other Race** | 1.45* | 1.07 | 1.28* | 1.74 | 1.27 | 2.9* | 1.46* | 1.69 | 1.02 |
| **Hispanic/Latino/Latina** | 0.91 | 0.98 | 0.86* | 0.95 | 0.9 | 1.19 | 0.59*** | 0.71 | 0.87 |
| **Urbanicity: Urban Cluster** | 0.88 | 1.32 | 0.92 | 1.46 | 1.16 | 1.3 | 0.98 | 1.25 | 1 |
| **Urbanicity: Rural** | 1.09 | 1.29* | 1.06 | 0.21 | 1.15 | 1.82 | 1.04 | 0.82 | 1.21* |

**p* < .05; ***p* < .01; ****p* <.001; *p*-values adjusted for multiple comparisons using Benjamini-Hochberg method.

**References**

[Garavan H, Bartsch H, Conway K, et al. Recruiting the ABCD sample: Design considerations and procedures.](https://www.zotero.org/google-docs/?iGPRh8)

[*Dev. Cogn. Neurosci*. 2018;32:16-22. doi:10.1016/j.dcn.2018.04.004](https://www.zotero.org/google-docs/?iGPRh8)

W.G. Iacono, A.C. Heath, J.K. Hewitt, M.C. Neale, M.T. Banich, M.M. Luciana, P.A. Madden, D.M. Barch, J.M. Bjork. The utility of twins in developmental cognitive neuroscience research: how twins strengthen the ABCD research design. *Dev. Cogn. Neurosci.* 2018; 32:30-42. doi: [10.1016/j.dcn.2017.09.001](https://doi.org/10.1016/j.dcn.2017.09.001)
